# Data on the chemical composition of Lake Onego water in 2019-2021

**DOI:** 10.1016/j.dib.2022.108079

**Published:** 2022-03-21

**Authors:** Mikhail Zobkov, Maria Zobkova, Natalia Galakhina, Tatiana Efremova, Natalya Efremenko, Natalia Kulik

**Affiliations:** Northern Water Problems Institute of the Karelian Research Centre of the Russian Academy of Sciences, 50 A. Nevskogo prospekt, Petrozavodsk, Karelia, 185030, Russia

**Keywords:** Surface water, Hydrochemistry, Nutrients, Organic matter, Heavy metals, Lake Onego

## Abstract

The present dataset contains the chemical parameters of Lake Onego water. The data were obtained in different seasons covering the period 2019-2021. The concentration of Na^+^ and Cl^−^ ions, the content of organic matter (TOC, COD_Mn_, COD_Cr_, water color, BOD_5_), nutrients (PO_4_-P, TP, NH_4_-N, NO_2_-N, NO_3_-N, TN), Fe, Mn, heavy metals (Cu, Ni, Cr, Zn, Cd, Pb), total suspended solids (TSS), conductivity, and pH of water were measured. The analyses were carried out by atomic absorption spectrometry, ICP-MS, spectrophotometric, spectrometric, gravimetric, flamephotometric, titrimetric methods, potentiometric, and conductometric determination. The data are useful for comparative analysis of the hydrochemical characteristics of Lake Onego and other large lakes, and they also allow to assess the water quality of the lake as a whole and in its particular individual areas.

## Specifications Table

**Table:** 

Subject area	Environmental science
More specific subject area	Hydrochemistry
Type of data	Table, figure
How data was acquired	Field sampling, chemical analysis
Data format	Raw
Description of data collection	The data were collected in different seasons of 2019-2021. Water was sampled from the surface (0.5 m) and near-bottom (1 m from the bottom) layers using a Niskin Bottle. The water depth and temperature were measured *in situ* using the CastAway probe (USA) at each station. In spring, summer, and autumn, some components (PO_4_-P, NO_2_-N, NH_4_-N, BOD_5_) were analyzed in the shipboard laboratory; other parameters (Na^+^ and Cl^−^ content, iron, manganese, total nitrogen, nitrates, total suspended solids, heavy metals (Ni, Cu, Pb, Zn, Cd, Cr), water color, pH, conductivity, TOC, COD_Mn_, COD_Cr_) were measured in the stationary laboratory. In winter, all analyses were performed in the stationary laboratory.
Data source location	Lake Onego, 35 stations, 230 samples, surface and near-bottom layers
Data accessibility	Repository name: Mendeley Data identification number: doi:10.17632/k4f59fyhmy.1 Direct URL to data: http://dx.doi.org/10.17632/k4f59fyhmy.1
Related research article	N. Galakhina, M. Zobkov, M. Zobkova, Current chemistry of Lake Onego and its spatial and temporal changes for the last three decades with special reference to nutrient concentrations, Environmental Nanotechnology, Monitoring & Management. 17 (2022) 100619. https://doi.org/10.1016/j.enmm.2021.100619.

## Value of the Data

• The chemical composition of Lake Onego water in different seasons of 2019-2021 is presented.
• The hydrochemical data presented in this article will enable society to understand the water quality of Lake Onego as it is the main source of freshwater.
• The obtained data allow tracing the distribution of contaminated waters originating from different sources.
• The obtained data can be used for comparative analysis of the hydrochemical characteristics of other large lakes. The database can also be applied to produce and verify mathematical models of chemical substances circulation in large lakes.

## 1 Data Description

**Fig. 1:**
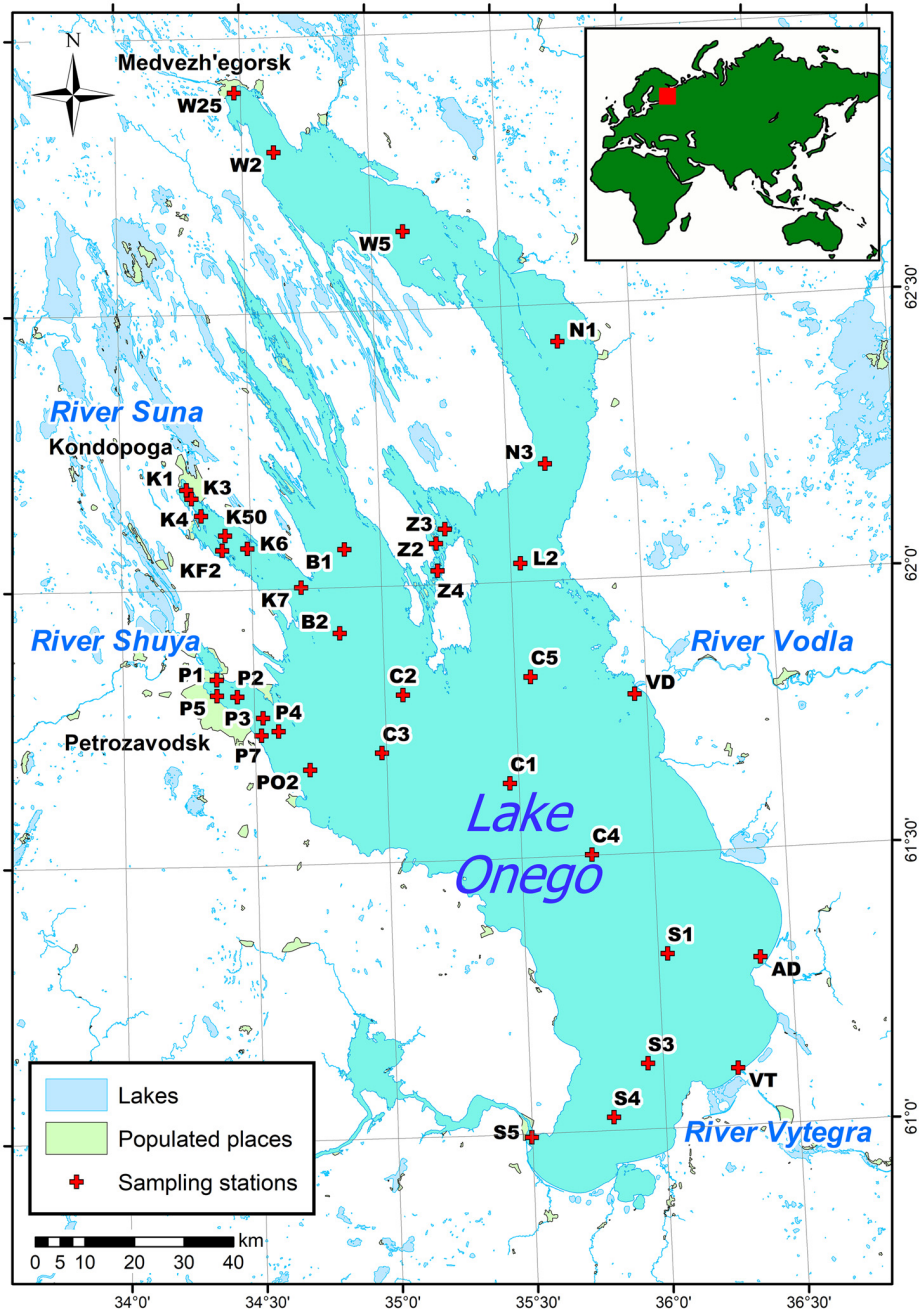
Sampling sites location.

The dataset contains information on the water chemical composition of Lake Onego in different seasons of 2019-2021. Water samples were collected using research vessels in spring, summer, and autumn. In winter, water samples were collected from the ice surface. The location of the sampling sites is presented in Fig. 1. Analytical methods commonly accepted in hydrochemical practice were used to determine the chemical parameters of water (Table 1). Detailed information is provided for each station of water sampling (station index, their geographic coordinates, sampling date, sampling depth, temperature, and water chemical composition). The data is provided in Microsoft Excel format.

**Table 1:** Chemical analysis methods.

Physico-chemical parameters, units	Analytical method	Detection limit	SD
рН	Potentiometric determination by glass electrode		4.0-10.0 – 0.05
Electric conductivity (EC), µS/cm	Determination by water conductivity meter Agilent 3200C at 25° C	1	4%
Na^+^, mg/L	Flamephotometric determination (Shimadzu AA 6200)	0.5	1.0-50 mg/L – 3%
Cl^−^, mg/L	Spectrophotometric determination with mercury thiocyanate and iron (III) nitrate, λ = 460 nm	0.1	0.2-2.0 mg/L – 0.035; 2.0-6.0 mg/L – 0.08; 6.0-10.0 mg/L – 0.23
Fe_,_ mg/L	FAAS (Shimadzu AA 6800)	0.05	0.1-1.0 mg/L – 11%; 1.0-15.0 mg/L – 7%
	GFAAS (Shimadzu AA 6800)	0.004	0.01-0.1 mg/L – 20%
Mn, µg/L	FAAS (Shimadzu AA 6800)	5	10-50 µg/L – 10%; 50-100 µg/L – 5%; 100-2,000 µg/L – 3%
	GFAAS (Shimadzu AA 6800)	0.04	1-15 µg/L – 10%
Heavy metals (Cu, Ni, Cr, Zn, Cd, Pb), µg/L	ICP-MS (Agilent 7500a)	Cu – 0.5 µg/L, Ni – 0.3 µg/L, Cr – 0.1 µg/L, Zn – 0.3 µg/L, Cd – 0.02 µg/L, Pb – 0.01 µg/L	10%
NH_4_-N, mgN/L	Spectrophotometric indophenol method with phenol and hypochlorite, λ=630 nm	0.005	0.02-0.05 mgN/L – 3.5%; 0.05-0.10 mgN/L – 2%; 0.10-0.20 mgN/L – 1%
NO_2_-N, mgN/L	Spectrophotometric method with sulfanilamide and N-(1-Naphthyl)-ethylenediamine-dihydrochloride, λ=543 nm	0.001	0.005-0.05 mgN/L – 1.2%; 0.05-0.3 mgN/L – 0.5%
NO_3_-N, mgN/L	Reduction to NO_2_^−^ with a Cd-reduction column and determination as NO_2_-N	0.003	0.005-0.10 mgN/L – 5%; 0.10-0.30 mgN/L – 2%
Total Nitrogen (TN), mg/L	Spectrometric method with potassium persulfate following mineralization in a thermostat to NO_3_^−^, λ=207 nm	0.04	0.05-2.0 – 4.5%
PO_4_-P, µgP/L	Spectrophotometric method with ammonium molybdate and ascorbinic acid reduction to phosphatomolybdic heteropolyacid, λ=882 nm	2	10-50 µgР/L – 1%; 20-200 µgР/L – 0,5%
Total Phosphorus (TP), µgP/L	Oxidation of organic matter by K_2_S_2_O_8_ in acidic media and determine PO_4_-P	5	20-40 µgР/L – 6.5%; 40-200 µgР/L – 3.5%
TOC, mg/L	Photochemical oxidation with ammonium persulfate	0.5	0.1 mgС/L
COD_Mn_,mg O/L	Titrimetric determination in acidic media (Kubel method)	0.25	5-50 mg O/L – 3%
COD_Cr_, mg O/L	Dichromate sulfuric acid oxidation of organic matter and titrimetric determination with ferroin	4	10-100 mg O/L – 7%
BOD_5_, mg O_2_/L	Determination by the closed bottle test	0.5	0.5-2.0 mg О_2_/L – 10%; 2.0-6.0 mg О_2_/L – 5%
TSS, mg/L	Gravimetric determination, 0.45 µm membrane filter	0.5	0.5-1.0 mg/L – 22%; 1.0-10 mg/L – 18%; 10-100 mg/L – 12%; 100-5000 mg/L – 9%; 5-50 g/L – 5%
Water color, mg Pt-Co/L	Photometric determination, λ=410 nm	5	up to 50 mg Pt-Co/L – 20%, over 50 mg Pt-Co/L - 10%

## 2 Materials and Methods

### 2.1 Study area description

Lake Onego is a large boreal lake situated in the north-western part of the Russian Federation and belongs to the Baltic Sea basin. Having a catchment area of 53100 km^2^ [1], a water volume of 295 km^3^, a mean depth of 30 m, a maximum depth – 120 m, and a surface area – 9720 km^2^ [2], it is the second largest lake in Europe after Lake Ladoga. The lake's basin is located on the Baltic crystalline shield in the north and the Russian Platform in the south [1]. Due to differences in geological structure, the northern and southern parts of the watershed differ in the share of lakes and wetlands, as well as in the water chemical composition of tributaries. As a large cold-water reservoir, the lake experiences water mixing twice a year in spring and autumn [3]. Spring heating initiates the formation of a thermal bar which separates the warmer coastal waters from colder homogeneous pelagic waters [3]. The tributaries of Lake Onego are 1,152 rivers, and the largest of them are rivers Shuya, Suna, and Vodla with about 60% of river discharge into the lake [1]. River discharge provides 73% of the water balance of Lake Onego [1], and less than 30% is accounted for by atmospheric precipitation, groundwater inflow, and wastewater discharge [4]. The River Svir', the largest tributary of Lake Ladoga, outflows Lake Onego.

The largest bays of Lake Onego are Petrozavodsk, Kondopoga, and Povenets, situated in the northern part of the lake. These bays are experiencing significant natural and anthropogenic influences. The River Shuya discharge (3.00 km^3^/year [1]), wastewater discharges of the Petrozavodsk industrial center (49-52 million m^3^/year), and storm runoff from Petrozavodsk are sources of contamination in the Petrozavodsk Bay [5]. The Kondopoga Bay receives the River Suna discharge (2.27 km/year [1]) and wastewater discharge of the Kondopoga Pulp and Paper Mill (PPM) with sulfite process of cellulose production and its volume is 50.7 million m^3^/year [6]. The Povenets Bay is influenced by untreated domestic water discharges from the town of Medvezh'egorsk (about 1.6 million m^3^/year) [7]. The river and wastewater discharges are the main sources of nutrients to Lake Onego [4]. Besides, Lake Onego is influenced by trout farms, most of which are located in the central part of the Kondopoga Bay. Intensively developing trout farming causes contamination of the water bodies with nutrients, which will lead to the development of local eutrophication zones [8] and, as a result, deterioration of water quality. Thus, the main anthropogenic sources are located in the largest bays of the lake as evidenced by the contamination of their waters.

### 2.2 Sample collection and analytical procedures

Water samples were collected in autumn 2019, spring and summer 2020 at Lake Onego (35 stations in its different regions), including the lake outflow (Svir' River) and in the estuarial areas of the rivers Shuya, Vodla, Andoma, and Vytegra, from RVs “Ecolog” and “Poseidon”. In winter 2021, samples were collected in the Kondopoga, Petrozavodsk bays and in the pelagic part of the Lake at station C3 from the ice surface. To determine most of the chemical characteristics, samples were collected at a depth of 0.5 m below the surface and a hight 1 m above the bottom with a Niskin Bottle. For heavy metal analysis, samples were collected in the same location and at the same depth with a polytetrafluoroethylene bathometer. The water depth and temperature were measured at each station *in situ* using the CastAway probe (USA). Water samples were collected in clean plastic vials. To determine the total phosphorus and iron content, water was collected in 250 mL polyethylene vials and then preserved with 4N H_2_SO_4_ solution (Reag. Ph.Eur., grade for analysis, ISO, Company Panreac Quimica S.L.U.). For Na^+^, Cl^−^, color, and TSS, determination subsamples were filtered with 0.45 µm membrane filters (47 mm diameter, Vladipor, Russia). Subsamples for heavy metal analysis were collected in 100 mL polyethylene flasks and instantly acidified with concentrated HNO_3_ (69% Suprapur®, Company Merck KGaA). The samples were stored in the dark at 4 °C before the analysis.

Chemical analyses were performed by corresponding methods (Table 1) in the Laboratory of Hydrochemistry and Hydrogeology, Northern Water Problems Institute of Karelian Research Center of the Russian Academy of Sciences. The reliability of the data is affirmed by the international cooperative program for the assessment and monitoring of the effects of air pollution on rivers and lakes [9].

### 2.3 Quality analysis and quality control

Quality analysis and quality control procedures were implemented according to [10]. The quality of the data obtained by each method of chemical analysis was regularly controlled using standards, blanks, and test samples. Reliability of the analysis was monitored using qualified standards as the standard deviation of repeatability. The intermediate precision was controlled using the studied samples. The control samples were analyzed similarly to the environmental samples, and one control (sample) was measured for every 15 environmental samples. The stability of the data was controlled in the range of the most common values or concentrations of the measured parameters. The standards were prepared using the state standard reference sample. Both single-component and multicomponent standards were used. In addition to the control measures listed above, the ICP-MS method uses the internal standard method to compensate and track instrumental drift and take into account changes in the analysis conditions. Individual samples for a number of elements (Na, Cu, Mn, Ni, Zn, Pb, Cd) were analyzed simultaneously by two methods (atomic absorption and ICP-MS) with convergence control. All measuring equipment was calibrated.

## Credit Authorship Contribution Statement

**Mikhail Zobkov:** Conceptualization, Methodology, Investigation, Writing - Review & Editing, Visualization, Supervision, Funding acquisition. **Maria Zobkova:** Investigation, Resources, Writing - Review & Editing. **Natalia Galakhina:** Data Curation, Writing – Original Draft. **Tatiana Efremova:** Investigation, Resources. **Natalya Efremenko:** Validation, Resources, Data acquisition. **Natalia Kulik:** Resources, Data acquisition.

## Declaration of Competing Interest

The authors declare that they have no known competing financial interests or personal relationships that could have appeared to influence the work reported in this paper.

## Data Availability

Lake Onego water chemical composition based on seasonal field surveys in 2019-2021 (Original data) (Mendeley Data).
